# Application of Deep Learning Model in the Sonographic Diagnosis of Uterine Adenomyosis

**DOI:** 10.3390/ijerph20031724

**Published:** 2023-01-18

**Authors:** Diego Raimondo, Antonio Raffone, Anna Chiara Aru, Matteo Giorgi, Ilaria Giaquinto, Emanuela Spagnolo, Antonio Travaglino, Federico Andrea Galatolo, Mario Giovanni Cosimo Antonio Cimino, Jacopo Lenzi, Gabriele Centini, Lucia Lazzeri, Antonio Mollo, Renato Seracchioli, Paolo Casadio

**Affiliations:** 1 Division of Gynecology and Human Reproduction Physiopathology, IRCCS Azienda Ospedaliero-Universitaria di Bologna, 40126 Bologna, Italy; 2 Department of Medical and Surgical Sciences (DIMEC), University of Bologna, 40126 Bologna, Italy; 3 Department of Molecular and Developmental Medicine, Obstetrics and Gynecological Clinic, University of Siena, 53100 Siena, Italy; 4 Department of Obstetrics and Gynecology, Morgagni–Pierantoni Hospital, 47100 Forlì, Italy; 5 Department of Obstetrics and Gynecology, Hospital Universitario La Paz, Paseo de la Castellana, 28046 Madrid, Spain; 6 Pathology Unit, Department of Woman and Child’s Health and Public Health Sciences, Fondazione Policlinico Universitario Agostino Gemelli IRCCS, 00168 Rome, Italy; 7 Pathology Unit, Department of Advanced Biomedical Sciences, School of Medicine, University of Naples Federico II, 80138 Naples, Italy; 8 Department of Information Engineering, University of Pisa, 56100 Pisa, Italy; 9 Department of Biomedical and Neuromotor Sciences, University of Bologna, 40126 Bologna, Italy; 10 Gynecology and Obstetrics Unit, Department of Medicine, Surgery and Dentistry “Schola Medica Salernitana”, University of Salerno, 84084 Baronissi, Italy

**Keywords:** artificial intelligence, deep learning, adenomyosis, endometriosis, trainee, ultrasound

## Abstract

Background: This study aims to evaluate the diagnostic performance of Deep Learning (DL) machine for the detection of adenomyosis on uterine ultrasonographic images and compare it to intermediate ultrasound skilled trainees. Methods: Prospective observational study were conducted between 1 and 30 April 2022. Transvaginal ultrasound (TVUS) diagnosis of adenomyosis was investigated by an experienced sonographer on 100 fertile-age patients. Videoclips of the uterine corpus were recorded and sequential ultrasound images were extracted. Intermediate ultrasound-skilled trainees and DL machine were asked to make a diagnosis reviewing uterine images. We evaluated and compared the accuracy, sensitivity, positive predictive value, F1-score, specificity and negative predictive value of the DL model and the trainees for adenomyosis diagnosis. Results: Accuracy of DL and intermediate ultrasound-skilled trainees for the diagnosis of adenomyosis were 0.51 (95% CI, 0.48–0.54) and 0.70 (95% CI, 0.60–0.79), respectively. Sensitivity, specificity and F1-score of DL were 0.43 (95% CI, 0.38–0.48), 0.82 (95% CI, 0.79–0.85) and 0.46 (0.42–0.50), respectively, whereas intermediate ultrasound-skilled trainees had sensitivity of 0.72 (95% CI, 0.52–0.86), specificity of 0.69 (95% CI, 0.58–0.79) and F1-score of 0.55 (95% CI, 0.43–0.66). Conclusions: In this preliminary study DL model showed a lower accuracy but a higher specificity in diagnosing adenomyosis on ultrasonographic images compared to intermediate-skilled trainees.

## 1. Introduction

Adenomyosis is a benign gynecological disease described by the presence of endometrial glands and stroma within the myometrium, as well as reactive hyperplasia and hypertrophy of the muscular layer [1]. Adenomyosis can cause symptoms like heavy menstrual bleeding, dysmenorrhea and infertility [2,3,4,5,6].

Pathological examination of myometrial specimen remains the gold standard for the diagnosis of adenomyosis and its estimated prevalence ranges from 21% to 36% among hysterectomized women. However, only a small, selected percentage of symptomatic women with adenomyosis undergoes hysterectomy, and the real prevalence of the disease is underestimated [3,7,8,9].

Transvaginal ultrasound (TVUS) represents the method of choice for the non-invasive diagnosis of adenomyosis with adequate sensitivity and specificity [10,11,12]. Standardization of terminology for the description of myometrium with Morphological Uterus Sonographic Assessment (MUSA) allowed universal recognition and assessment of typical adenomyotic ultrasound features [2,3,13].

Despite being low cost and easily accessible, TVUS has some limitations for the diagnosis of adenomyosis. It is an operator-dependent technique with adequate diagnostic performance and inter-operator reproducibility only if performed by expert sonographers [10,14,15]. In particular, Rasmussen et al. observed a moderate intra-operator agreement and a poor inter-operator agreement among medium experienced raters for the diagnosis of adenomyosis [14]. Therefore, expert sonographers in dedicated centers are recommended for the diagnosis of adenomyosis [9], with an overall TVUS sensibility and specificity of 81% and 87%, respectively [12].

Recently, the need to improve efficiency in all clinical settings using technological advances led to the development of powerful instruments such as artificial intelligence (AI) [16]. AI is defined as the use of several complex algorithm-based applications that can solve problems by simulating human cognitive functions, including data learning and processing, problem solving and decision making [17]. Machine learning (ML) and deep learning (DL) can be accounted among the newest developed technologies in this area. DL is a subfield of machine learning, able to consistently add new data with self-learning ability, thus increasing the performance of the application itself and able to find correlations that humans cannot [18].

AI applicability for healthcare purposes has already been partially investigated and has shown promising results in several medical fields, including gynecology. In particular, AI in gynecological studies was tested for several tasks on medical images, including discriminating malignancy or benignity of ovarian masses, diagnosing cervical cancer, staging endometrial cancer, or diagnosing rectosigmoid endometriosis [17,19,20].

To the best of our knowledge, no study has ever assessed the accuracy of DL in the diagnosis of adenomyosis using TVUS. Therefore, the aims of this study are to first evaluate the diagnostic performance of DL in the diagnosis of adenomyosis on uterine ultrasonographic images and compare it to that of intermediate ultrasound skilled trainees.

## 2. Materials and Methods

### 2.1. Study Protocol and Selection Criteria

This was a proof-of-concept, monocentric, observational, cross-sectional study, conducted in a tertiary academic centre. The whole study followed an a priori protocol previously drawn up according to STROBE guidelines and checklist [21].

Exclusion criteria were as follows: age less than 18 years old, virgo intacta status, ongoing or recent (less than 6 months) pregnancy, suspicion of gynecological malignancy, previous hysterectomy, menopausal status, coexistence of adenomyosis and fibroids at TVUS. From 1 to 30 April 2022, all eligible consecutive women referring to our tertiary gynecological ultrasound clinic were consecutively asked to participate to the study.

### 2.2. Study Outcomes

Accuracy was used as the primary evaluation metric for the diagnostic performance of DL machine in the diagnosis of adenomyosis at 2D gray scale mode TVUS images. Accuracy is a statistical measure of how well a binary classification test correctly identifies or excludes a condition, that is, the proportion of correct predictions (both true positives and true negatives) among the total number of patients examined.

Other metrics used to measure accuracy from different perspectives were the following: recall (sensitivity), precision (positive predictive value, PPV), F1-score (harmonic mean of precision and recall), specificity and negative predictive value (NPV). The same metrics were used to assess the performance of intermediate skilled trainees for the diagnosis of adenomyosis and were informally compared with those obtained with DL machine.

Secondarily, all the measures listed above were calculated using the diagnoses of fibrosis and homogeneous echogenicity as reference.

### 2.3. Patient Assessment

For each patient, anamnestic and clinical data were acquired as follows: age, body mass index (BMI), parity, history of infertility, previous endometriosis surgery, moderate-to-severe pain symptoms defined as numerical rating scale (NRS) equal or superior to 5 [22], heavy menstrual bleeding referred to as pictorial blood loss analysis chart ≥100 [23] and use of hormonal therapy.

### 2.4. Ultrasound Details

Voluson E8 ultrasound machine (GE Healthcare, Zipf, Austria) with a 4–9 MHz volumetric vaginal probe was used for all acquisitions. Ultrasound scans were obtained with patients in a modified lithotomic position. During the 2D gray scale mode TVUS examination, an expert sonographer classified uteruses in three groups: homogenous myometrial echogenicity, fibroids or adenomyosis.

Adenomyosis was diagnosed when two or more of the following sonographic criteria were present: globular uterus appearance, asymmetrical thickening, hypoechogenic myometrial cysts, hyperechoic islands, fan-shaped shadowing, echogenic subendometrial lines and buds, junctional zone irregolarities [2,3,8]. Otherwise, uterine fibroids were diagnosed as well-defined round lesions of the myometrium, frequently with shadows at the edge or an internal fan-shaped shadow [2,24].

For each patient, presence of deep endometriotic lesions and endometrioma was also investigated according to IDEA consensus [25,26].

### 2.5. Deep Learning (DL)

An end-to-end DL model was developed for the classification of uterine images. Sequential ultrasound images including uterine corpus and cervix were extracted from ultrasound video clips by an automatic system. Manual segmentation was performed by the experienced sonographer. Uterine boundaries were manually traced in the sagittal scan including a region of interest (ROI) that clearly highlighted ultrasound features of adenomyosis or fibroids, according to literature. These ultrasound images were used for the construction, validation and testing of the DL system. 

The available dataset of 100 ultrasound video clips was divided in a random and balanced way into three parts: training (*n* = 30), validation (*n* = 30) and testing set (*n* = 40). The training set was used to train the network by teaching it the parameters of the models. Two architectures were considered: ResNet and Vgg. Among these, Vgg13, Vgg19, ResNet 18 and ResNet 34 models were used.

The validation set was used for early stopping, which saves the network weights at the point of best performance, and for optimizing the hyper-parameters. The hyper-parameters used were as follows:

- Pre-trained and un-trained networks on Microsoft Common Objects in Context;
- Batch size: 8-16-32;
- Patience: 3-5-10.

L1 and L2 regularizations have been implemented in the network.

To find the best combination of hyperparameters, Tree Parzen Estimator (TPE) was used as a sampler and Successive Halving Pruner (SHP) as a pruner. To reduce over-fitting, data augmentation was also applied, which generates additional training models using random image transformations. To this end, the captured images were extracted with the resolution reduced from 300 × 300 pixels to 224 × 224 pixels, random horizontal flips and vertical flips were employed and Gaussian blur was applied.

The test set was used to independently assess the generalization error for the final models chosen.

Diagnostic performance of each DL models was acquired.

### 2.6. Diagnostic Performance of Trainees 

For each patient, uterine images were acquired for storage using short video clips (8–10 s). The uterus (cervix and corpus) was filmed in a sagittal plane and with a lateral left-to-right movement of the probe, using a grayscale mode. Videoclips were downloaded in Mp4 format from the hospital image database system and then de-identified prior to being reanalyzed by three intermediate ultrasound skilled trainees. These trainees were 4th year residents in O&G with intermediate ultrasound skills (consisting of more than 500 gynecologic ultrasound cases) doing their postgraduate studies in endometriosis management [27]. The trainees blinded to clinical data were asked separately to make their own diagnosis reviewing uterine images of testing set. Diagnostic performance of each trainee was acquired.

### 2.7. Statistical Analysis

Numerical variables were summarized as mean ± SD or median (95% CI); categorical variables were summarized as counts and percentages. Chi-squared test, Fisher’s exact test and variance analysis were used for comparison of categorical and numerical variables, where appropriate.

To compare the performance of the best DL model with that of the best trainee in diagnosing adenomyosis, accuracy, sensitivity, positive predictive value (PPV), F1-score (harmonic mean of positive predictive value and sensitivity), specificity and negative predictive value (NPV) were calculated.

Analyses were conducted using Stata 15 software (StataCorp. 2017. Stata Statistical Software: Release 15. College Station, TX, USA: StataCorp LLC). The significance level was set at 5%.

### 2.8. Ethical Statement and Informed Consent

The study protocol received approval by the local Ethics Committee (114/2022/Oss/AOUBo). All patients signed an informed consent before entering the study, and all data were anonymized.

## 3. Results

During the study period, 100 eligible patients were enrolled. Ultrasound diagnosis by expert operator were as follows: 45 patients with homogeneous echogenicity of the myometrium, 30 with fibroids and 25 women with adenomyosis.

Baseline and clinical characteristics are summarized in Table 1. Mean (±SD) age and BMI of the study sample were 35.4 ± 8.0 years and 22.5 ± 2.5 kg/m^2^, respectively. There was no significant difference in terms of baseline data among the three study groups, except for age, rate of spontaneous delivery and heavy menstrual bleeding, which were higher in the fibroids group, while previous surgery for endometriosis was more frequent in the adenomyosis group.

Sonographic signs suggestive for adenomyosis in the “adenomyosis group” diagnosed by expert operator are reported in Table 2. “Globular uterus” was the most frequent sonographic sign (72%), followed by “asymmetrical thickening” and “fan shaped shadowing” (60%).

After the application of data augmentation, number of uterine images were as follows:

- Training set: *n* = 1645 homogeneous echogenicity, *n* = 1071 fibroids, *n* = 836 adenomyosis;
- Validation set: *n* = 481 homogeneous echogenicity, *n* = 336 fibroids, *n* = 252 adenomyosis;
- Testing set: *n* = 495 homogeneous echogenicity, *n* = 359 fibroids, *n* = 336 adenomyosis.

**Figure 1:**
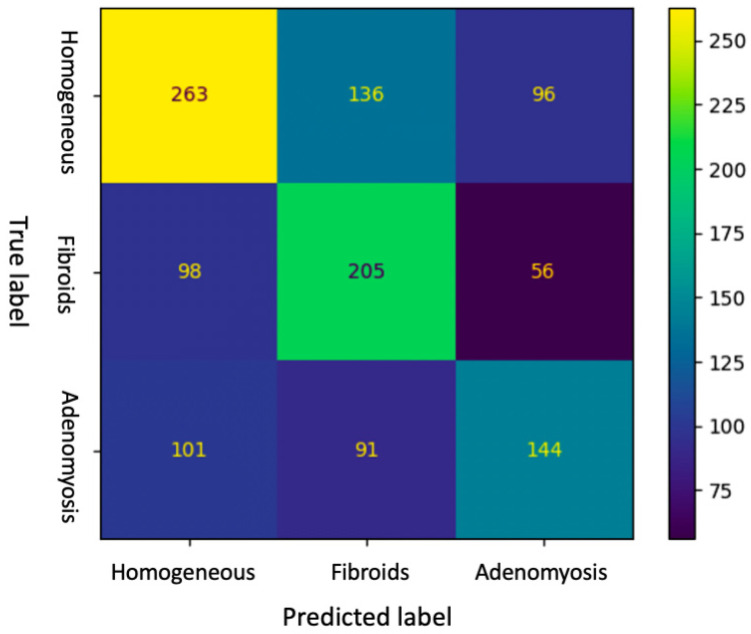
Confusion matrix of the DL diagnosis of the testing set.

Confusion matrix of the DL for the testing set is shown in Figure 1. The matrix highlights where the model fails. Rows show diagnosis made by the experienced sonographer (true label), while columns show predictions made by the machine (predicted label). Diagonal elements were the number of points where the predicted label was the same as the actual label, while the off-diagonal ones were misinterpreted by the model.

As reported in Table 3, accuracy of DL and intermediate ultrasound-skilled trainees for the diagnosis of adenomyosis were 0.51 (95% CI, 0.48–0.54) and 0.70 (95% CI, 0.60–0.79), respectively. Sensitivity, specificity and F1-score of DL were 0.43 (95% CI, 0.38–0.48), 0.82 (95% CI, 0.79–0.85) and 0.46 (0.42–0.50), respectively, whereas intermediate ultrasound-skilled trainees had sensitivity of 0.72 (95% CI, 0.52–0.86), specificity of 0.69 (95% CI, 0.58–0.79) and F1-score of 0.55 (95% CI, 0.43–0.66). Positive predictive value and negative predictive value for the diagnosis of adenomyosis were 0.49 (95% CI, 0.43–0.55) and 0.78 (95% CI, 0.75–0.81) for DL, and 0.44 (95% CI, 0.30–0.59) and 0.88 (95% CI, 0.77–0.94) for trainees, respectively.

Regarding fibroids diagnosis, DL model reached sensitivity, specificity and F1-score of 0.57 (95% CI, 0.52–0.62), 0.73 (95% CI, 0.70–0.76) and 0.52 (0.48–0.56), respectively, with an accuracy of 0.68 (95% CI, 0.65–0.71). Intermediate ultrasound skilled trainees had sensitivity of 0.63 (95% CI, 0.46–0.78), specificity of 0.82 (95% CI, 0.72–0.89), F1-score of 0.61 (95% CI, 0.49–0.72) and accuracy of 0.77 (95% CI, 0.67–0.84). Positive predictive value and negative predictive value for the diagnosis of fibroids were 0.47 (0.42–0.52) and 0.80 (0.77–0.83) for DL, and 0.61 (0.43–0.76) and 0.84 (0.74–0.91) for trainees, respectively.

## 4. Discussion

Despite AI recently gaining popularity in the field of medical imaging and has experienced increased its applications in gynecology, no study has ever used this tool in the diagnosis of uterine adenomyosis. Therefore, this study can be considered a proof-of-concept for this issue.

Recently, DL based on artificial neural networks with representation learning has been adopted to help operators to untangle among differential diagnoses.

In the present study, the DL model showed a low accuracy in the diagnosis of uterine adenomyosis (51%). This observation may reflect the complexity of the disease. Indeed, adenomyosis is a heterogeneous disease that can have several phenotypes, varying per extension (diffuse, focal or adenomyoma) and location (internal myometrial or junctional invasion) within the myometrium [4,11].

As secondary finding, the accuracy of intermediate ultrasound-skilled trainees (70%) resulted higher than that of the DL. Moreover, these trainees showed a higher sensitivity (72%) but a lower specificity (69%) compared to those of the DL. This over-diagnosis could be explained by the tertiary center setting in which frequency of adenomyosis is estimated higher than general population and the offline assessment of uterine images instead of personal execution of TVUS. Conversely, the DL model showed a higher specificity, being more effective in identifying healthy uteruses, with low false positive values. Indeed, the DL model could be a useful tool to exclude adenomyosis where it is not present and disprove the over diagnosis of less experienced operators, avoiding unnecessary second-level examinations or over treatment cases.

Limitations of our study are the small sample size and the monocentric design, reducing the generalizability of our results. Larger multicentric studies are needed to better evaluate the potential clinical aid of AI in the diagnosis of adenomyosis. Although the lack of histological confirmation of adenomyosis may be considered another limitation of the study, pathological examination is unethical in patients without any indication for surgery. On the other hand, to date, an experienced sonographer must be considered an adequate alternative to histological diagnosis in women who are asymptomatic or have not completed their reproductive plan.

The impossibility to fully investigate the JZ by using 3D-TVUS examination and to evaluate translesional vascularity through Power Doppler mode may have influenced the diagnostic performance of the expert sonographer firstly and then that of the trainees and the DL machine.

In order to improve the diagnostic performance of the DL in diagnosing adenomyosis, future research can be focused to specific training of the DL machine on the recognition of each of ultrasound criteria suggestive for adenomyosis. Moreover, more studies are needed to evaluate any improvement of DL performance, adding other sonographic signs (i.e., translesional vascularity using Power Doppler and junctional zone thickness or irregularities at 3D TVUS) and/or clinical data (i.e., presence and severity of pain symptoms and uterine tenderness).

## 5. Conclusions

In this proof-of-concept study, the DL model achieved a low diagnostic performance for the detection of adenomyosis with accuracy of 51%, lower than that of intermediate skilled trainees. Sensitivity and F1-score of the intermediate skilled trainees were higher than those of DL as well. However, DL model showed potential for excluding adenomyotic uteri, with higher specificity and NPV than those of intermediate skilled trainees.

Larger multicentric studies with adjuvant investigation of JZ by 3D-TVUS and translesional vascularity through Power Doppler are needed to better evaluate the potential clinical application of AI in the diagnosis of adenomyosis.

## Figures and Tables

**Table 1:** Baseline characteristics of the study sample and of the three types of diagnosis by expert sonographers.

Variable	All (*n* = 100)	Diagnosis by Expert Sonographers			*p*-Value
		Adenomyosis	Fibroids	Homogenous Echogenicity	
		(*n* = 25)	(*n* = 30)	(*n* = 45)	
Age, years	35.4 ± 8.0	35.1 ± 7.1	42.1 ± 6.2	31.1 ± 6.5	<0.001 *
BMI, kg/m^2^	22.5 ± 2.5	22.0 ± 1.8	22.9 ± 2.3	22.5 ± 2.9	0.395
Spontaneous delivery	24 (24%)	5 (20%)	12 (40%)	7 (16%)	0.045 *
Caesarean section	9 (9%)	2 (8%)	4 (13%)	3 (7%)	0.608
Infertility	22 (22%)	4 (16%)	7 (23%)	11 (24%)	0.700
Previous surgery for endometriosis	21 (21%)	9 (36%)	7 (23%)	5 (11%)	0.046 *
Hormonal therapy	37 (37%)	14 (56%)	10 (33%)	13 (29%)	0.070
Moderate to severe pain symptoms (NRS equal or superior to 5)					
Dysmenorrhea	21 (21%)	8 (32%)	2 (7%)	11 (24%)	0.053
Chronic pelvic pain	8 (8%)	2 (8%)	1 (3%)	5 (11%)	0.574
Dyspareunia	19 (19%)	9 (36%)	4 (13%)	6 (13%)	0.063
Heavy menstrual bleeding	11 (11%)	3 (12%)	8 (27%)	0 (0%)	<0.001 *
Coexistence of endometriosis at TVUS					
Deep endometriosis	11 (11%)	4 (16%)	3 (10%)	4 (9%)	0.655
Endometrioma	27 (27%)	9 (36%)	7 (23%)	11 (24%)	0.501

* *p*-value ≤ 0.05. All characteristics were expressed as number (percentage). Abbreviations: BMI: body mass index; kg, kilograms; m, meters; NRS: numerical rating scale; TVUS: transvaginal ultrasound.

**Table 2:** Sonographic features of adenomyosis in the adenomyosis group (25 patients), assessed by an expert sonographer.

Characteristics	Prevalence *n* (%)
Globular uterus	18 (72%)
Asymmetrical thickening	15 (60%)
Fan-shaped shadowing	15 (60%)
Myometrial Cysts	12 (48%)
Junctional zone irregularities	8 (32%)
Hyperechoic islands	7 (28%)
Echogenic subendometrial lines and buds	7 (28%)
Question mark sign	7 (28%)

All characteristics were expressed as number (percentage).

**Table 3:** Diagnostic performance of intermediate ultrasound skilled trainees and DL in the diagnosis of homogeneous echogenicity, fibroid and adenomyosis in the testing set.

Variable	Adenomyosis		Fibroids		Homogeneous Echogenity	
	Intermediate Ultrasound Skilled Trainees	DL	Intermediate Ultrasound Skilled Trainees	DL	Intermediate Ultrasound Skilled Trainees	DL
Sensitivity	0.72 (0.52–0.86)	0.43 (0.38–0.48)	0.63 (0.46–0.78)	0.57 (0.52–0.62)	0.58 (0.44–0.72)	0.53 (0.49–0.57)
Specificity	0.69 (0.58–0.79)	0.82 (0.79–0.85)	0.82 (0.72–0.89)	0.73 (0.70–0.76)	0.84 (0.74–0.94)	0.71 (0.68–0.74)
PPV	0.44 (0.30–0.59)	0.49 (0.43–0.55)	0.61 (0.43–0.76)	0.47 (0.42–0.52)	0.74 (0.59–0.89)	0.57 (0.52–0.62)
NPV	0.88 (0.77–0.94)	0.78 (0.75–0.81)	0.84 (0.74–0.91)	0.80 (0.77–0.83)	0.71 (0.60–0.82)	0.68 (0.65–0.71)
Accuracy	0.70 (0.60–0.79)	0.51 (0.48–0.54)	0.77 (0.67–0.84)	0.68 (0.65–0.71)	0.72 (0.63–0.81)	0.64 (0.61–0.67)
F1-score	0.55 (0.43–0.66)	0.46 (0.42–0.50)	0.61 (0.49–0.72)	0.52 (0.48–0.56)	0.65 (0.52–0.78)	0.70 (0.67–0.73)

Values are expressed as median (95%, CI). Abbreviations: PPV, positive predictive value; NPV, negative predictive value; DL, deep learning.

## Data Availability

The data presented in this study are available on request from the corresponding author. The data are not publicly available due to the need for privacy maintenance of patients.
